# Mapping Physiotherapy Approaches for Stroke Survivors in Catalonia: A Cross-Sectional Study

**DOI:** 10.31083/RN37316

**Published:** 2025-06-16

**Authors:** Maria Masbernat-Almenara, Selma Peláez Hervás, Helena Fernández-Lago, Samira Gonzalez-Hoelling, Carina Salgueiro, Rosa Cabanas-Valdés

**Affiliations:** ^1^Department of Nursing and Physiotherapy, University of Lleida, 25198 Lleida, Spain; ^2^Research Group of Health Care (GReCS), Biomedical Research Institute of Lleida, Dr. Pifarré Foundation (IRBLleida), 25198 Lleida, Spain; ^3^Consolidated Research Group: Society, Health, Education and Culture (GESEC), University of Lleida, 25001 Lleida, Spain; ^4^Rehabilitation Service (ICEMEQ), Hospital Clínic de Barcelona, ​​08036 Barcelona, ​​Spain; ^5^Hospital Sociosanitari Mutuam Girona Neurorehabilitation Department, 17007 Girona, Spain; ^6^Department of Physiotherapy, Faculty of Medicine and Health Science, Universitat Internacional de Catalunya, 080195 Barcelona, Spain

**Keywords:** conventional physiotherapy, physiotherapy, questionnaire, observational study, rehabilitation, stroke, fisioterapia convencional, fisioterapia, cuestionario, estudio observacional, rehabilitación, ictus

## Abstract

**Background:**

Stroke is a leading cause of death and disability worldwide, prompting significant interest in rehabilitation. Despite existing recommendations and clinical guidelines, the current state of stroke rehabilitation practices in Catalonia remains unclear. This study aims to identify physiotherapists' main approaches for stroke survivors in Catalonia across recovery stages.

**Methods:**

An observational study was conducted via an anonymous survey distributed among all the registered members of the College of Physiotherapists of Catalonia (CPC). A total of 118 physiotherapists from both the public and private sector participated. The study collected data on therapists' experience, preferred therapeutic modalities, user demographics, and work settings. The data was collected from January to March, 2023.

**Results:**

The survey on stroke rehabilitation approaches showed that 57.60% of participants use a mix of methods (Basal Stimulation, Proprioceptive Neuromuscular Facilitation (PNF), neurodevelopmental or neurocognitive therapy) tailored to individuals or stroke stages, regardless of the work setting. Regarding the techniques, the most used were passive mobilization, stretching, task-oriented approaches, motor imagery, intensive therapy, mirror therapy, and balance training. In contrast, electrotherapy, music therapy, mindfulness, and advanced technologies were the least used.

**Conclusions:**

Physiotherapists did not rely on a single technique or approach; instead, they combined various methods. Therefore, we are unable to definitively determine what constitutes conventional physiotherapy. Considering this ambiguity, it is recommended to explicitly identify the techniques and methods used during conventional physical therapy in all scientific studies.

**Clinical Trial Registration:**

No: NCT05546840. 15 September 2022, https://clinicaltrials.gov/study/NCT05546840?cond=NCT05546840&rank.

## Abstract (es)

**Antecedentes:**

El ictus es una de las principales causas de muerte y discapacidad en todo el mundo, lo que suscita un gran interés en la rehabilitación. A pesar de las recomendaciones y guías clínicas existentes, el estado actual de la práctica de rehabilitación del ictus en Cataluña sigue sin estar claro. Este estudio tiene como objetivo identificar los principales enfoques de los fisioterapeutas para los supervivientes de ictus en Cataluña a través de las etapas de recuperación.

**Métodos:**

Se realizó un estudio observacional a través de una encuesta anónima distribuida entre todos los colegiados del Col.legi de Fisioterapeutes de Catalunya (CFC). Participaron 118 fisioterapeutas del sector público y privado. El estudio recogió datos sobre la experiencia de los terapeutas, las modalidades terapéuticas preferidas, los datos demográficos de los usuarios y los entornos de trabajo. Los datos se recogieron entre enero y marzo de 2023.

**Resultados:**

La encuesta sobre los enfoques de rehabilitación del ictus mostró que el 57,8% de los participantes utilizan un combinado de métodos (Estimulación Basal, Facilitación Neuromuscular Propioceptiva (FNP), terapia del neurodesarrollo o neurocognitiva) adaptados a los individuos o a las etapas del ictus, independientemente del entorno de trabajo. En cuanto a los enfoques, las más utilizadas fueron la movilización pasiva, los estiramientos, el enfoque orientado a tareas, la imaginería motora, la terapia intensiva, la terapia de espejo o el entrenamiento del equilibrio. Por el contrario, la electroterapia, la musicoterapia, el mindfulness o las tecnologías avanzadas fueron las menos utilizadas.

**Conclusiones:**

Los fisioterapeutas no se basan en una única técnica o enfoque, sino que combinan varios métodos. Por lo tanto, no podemos determinar de forma definitivaqué incluye la fisioterapia convencional. Teniendo en cuenta esta ambigüedad, se recomienda identificar explícitamente las técnicas y métodos utilizados como fisioterapia convencional en cada estudio científico.

**Registro de Ensayo Clínico:**

N.º: NCT05546840. 15 de septiembre de 2022, https://clinicaltrials.gov/study/NCT05546840?cond=NCT05546840&rank.

## 1. Introduction

Stroke is a significant global health concern, standing as one of the leading causes of death and disability [1, 2]. The prevalence of stroke affects a staggering 12.2 million people worldwide, with 100 million people living with stroke sequelae [3]. The burden of it amounts to around 800 billion euros [3]. About 113,000 people in Europe experience strokes annually [1, 4]. In Catalonia, a region of Spain, approximately 11,100 people were affected by stroke in 2021, accounting for a prevalence of 2.3% [5]. Stroke survivors often experience several different motor and sensory impairments that significantly affect their mobility and independence in performing activities of daily living [6]. Motor impairments include weakness or paralysis on one side of the body (hemiparesis or hemiplegia), coordination difficulties and balance, and challenges with fine and gross motor skills [7]. Sensory impairments can also be prevalent, affecting a stroke survivor’s ability to perceive touch, temperature, and spatial awareness. All of the above underscores the immense impact of stroke, prompting extensive research across various domains, including motor rehabilitation [7], which is a crucial element of the stroke care pathway for people with persisting movement and mobility deficits [8].

According the latest actualization of the Catalan Stroke Clinical Practice Guide, in 2004, stroke was the most common cause of neurorehabilitation in Catalonia (Spain) representing 1.1 % of hospital care and 16% of home rehabilitation [9]. People with stroke begin rehabilitation during the acute phase in the hospital. After medical stabilization, they may do inpatient rehabilitation or in specialized centers. Upon returning home, they can pursue rehabilitation through ambulatory care, home care, or at a physiotherapy center [9].

A new rehabilitation plan was developed in Catalonia in 2022, which sought to guide a comprehensive approach in treating the most prevalent diseases in Catalonia, including stroke [10]. This plan recommends intervention techniques based on scientific evidence, focusing on International Classification of Functioning, Disability and Health (ICF) dimensions [10]. This recommendations contrast with earlier Catalan (2007) and Spanish (2009) guides which categorized rehabilitation techniques into compensatory, facilitation, and modern techniques [9, 11].

Nowadays, the methods and techniques employed in physiotherapy for people with stroke can vary based on practitioners’ knowledge, experience, clinical practices, and personal preferences [12]. Several studies that addressed this variability have concluded that a consensus regarding rehabilitation is necessary because there is a lack of comprehensive understanding of physiotherapists’ currents practices [13, 14, 15]. In addition, in randomized controlled trials (RCTs), researchers often compare specific techniques to conventional therapy or usual care [16]. Moreover, there exists a variety of terminology referring to this counterpart, such as usual care, the standard of care, conventional therapy, or current clinical practice, that researchers have studied and tried to define in many reviews. Still these terms are widely under described or poorly referred into a guideline, all of this implying a lack of validity, generalizability, and interpretability of results [16, 17, 18]. Hence, understanding these terms is necessary.

In stroke rehabilitation, the term “conventional” physical therapy, encompassed within the broader umbrella of “usual care”, is widely used [17]. However, there is a lack of standardization of what “conventional” includes. A recent revision found that it covers different therapies, doses, and interventions, and only a tiny percentage of the included studies justified their intervention based on clinical practice guidelines [17]. For this reason, standardized “conventional physiotherapy” is needed following current clinical guidelines to uniform the rehabilitation protocols and facilitate experimental treatment replication [17].

For these reasons, the primary objective of this study is to identify the most used physiotherapy approaches, methods, and techniques in both the public and private healthcare settings. The second objective is to explore what is the conventional physiotherapy employed in the early rehabilitation phase, the late rehabilitation phase, and rehabilitation in chronic phase [19] stroke survivors in Catalonia.

## 2. Materials and Methods

### 2.1 Study Design

A descriptive cross-sectional study conducted in Catalonia, Spain between January, and March 2023, registered in ClinicalTrials.gov (https://clinicaltrials.gov/study/NCT05546840?cond=NCT05546840&rank). The study followed the STROBE guidelines, the STROBE checklist is included in the the **Supplementary Material 1** [20].

### 2.2 Setting and Participant Recruitment

The study was conducted in Catalonia, Spain, looking for those physiotherapists who carry out their activity either in the public system or private sector, including insurance companies. According to the General Register of Health Centers, Services and Establishments, there are currently 1774 physiotherapy centers in Catalonia, of which 47 are public [21].

According to the College of Physiotherapists of Catalonia (CPC), there are currently 15,000 members. In Spain, physiotherapy specializations are not officially recognized by the Consejo General de Colegios de Fisioterapeutas de España, neither in terms of defined guidelines to consider a professional specialized in a field of knowledge, nor the levels of expertise. This complex scenario makes difficult to determine the exact number of active physiotherapists working with neurological patients. On the CPC website there are 239 centers identified as neurological centers with working neurological expert physiotherapists. Due to this situation, the sampling strategy was non-probabilistic convenience. The CPC initiated the recruitment, which sent an email containing the survey link to all registered physiotherapists to ensure that only Catalan registered physiotherapists respond, and that each physiotherapist provides only one response. Inclusion criteria for the participants were physiotherapists registered in the CPC who currently treat at least one stroke patient per week.

### 2.3 Data Collection

Data were collected between January and March 2023. The questionnaire was designed ad hoc combining open and closed questions with response options (**Supplementary Material 2**). The questionnaire consisted of three blocks: (i) Demographic data, specific training in neurology, years of experience in Neurorehabilitation, work setting and post-stroke rehabilitation phases; (ii) Therapeutic post-stroke intervention (physiotherapy techniques, method approach in physiotherapy); (iii) Open question about what conventional physiotherapy is. To avoid missing data all the questions were mandatory. The results were exported to Microsoft Excel Version 16.0. (Microsoft Corporation., Redmond, WA, USA).

### 2.4 Data Analysis

Data obtained in this study were analyzed with IBM SPSS Statistics for Windows (Version 27) (SPSS Inc., Chicago, IL, USA). A descriptive analysis was performed (frequency and percentages of the variables). The independence chi-square test and Fisher exact test were used to look for associations between the variable specific approach and non-specific approach with phases, settings, and years of experience, with a statistical significance of 5%. The mentioned variables were coded to perform chi-square test and Fisher exact test.

### 2.5 Rigor

A team of experts with over ten years of experience created the questionnaire, comprising clinicians and researchers specialized in stroke and neurorehabilitation. The experts conducted brainstorming sessions through various rounds of focus groups to design the questionnaire and reached a consensus regarding the most critical questions for the research. To avoid any bias, the final draft was sent to five physiotherapists outside the study to give their opinions on its ease of understanding. With their feedback, we designed the final questionnaire. The source of material used and relevant ethical framework for all experiments should be clearly identified (ethics approval and/or written informed consent). Methods already published should be indicated by a reference: only relevant modifications should be described. This implies that a full description of all the experiments described in Results and presented in the Figures/Tables is expected in this section. For each experiment, all steps need to be mentioned, along with instruments the analyses were performed on, reagents and methods to permit the replication of the work by others. We would encourage authors to submitting a detailed Bio-protocol.

## 3. Results

A total of 118 physiotherapists from Catalonia (87 women, 30 men, one no binary), aged between 21 and 65, with professional experience in neurorehabilitation ranging from one to more than ten years in different settings (public or private sector) and from various regions of Catalonia (Lleida, Tarragona, Barcelona, and Girona), answered the survey (Table 1).

**Table 1.** **Demographic and professional data**.

N = 118		N	Percentage
Sex			
	Female	87	73.7%
	Male	30	30%
	No binary	1	0.8%
Age			
	Between 21 and 30 years	39	33.1%
	Between 31 and 40 years	38	32.2%
	Between 41 and 50 years	30	25.4%
	Between 51 and 65 years	11	9.3%
Region			
	Barcelona	86	72.9%
	Girona	13	11%
	Lleida	8	6.8%
	Tarragona	11	9.3%
Training in Nhb			
	Continuing education courses	55	46.6%
	Specific graduate program	20	16.95%
	Specialized master’s program	33	28%
	PhD program	5	4.23%
Years of experience in Nhb			
	0–2 years	21	17.8%
	3–5 years	29	24.6%
	6–9 years	19	16.1%
	More than 10 years	49	41.5%
Work settings			
	Public	35	29.7%
	Private	56	47.5%
	Public and private	27	22.9%

Nhb, neurorehabilitation.

### 3.1 Therapeutic Intervention

We classified the responses as related to the therapeutic intervention at different points.

#### 3.1.1 Descriptive Analysis of the Use of Physiotherapy Techniques

**Fig. 1.**
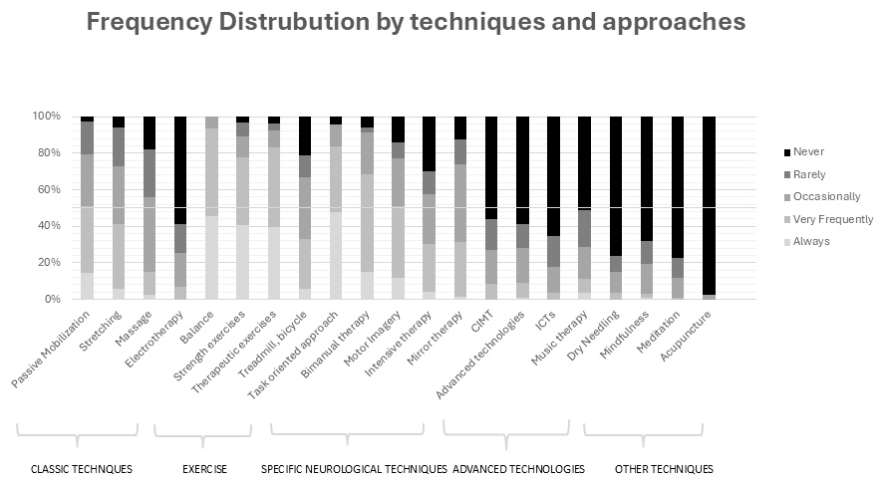
**This graph shows the distribution of participants’ responses based on the techniques they utilized in stroke rehabilitation**. CIMT, constraint-induced movement therapy; ICTs, information and communication technologies.

The respondents qualified the different approaches using of frequencies (always, very frequently, occasionally, rarely, and never). Among the classic techniques included, the most used was passive mobilization, with 14.4% of the participants indicating they always used it, and 36% using it very frequently, while electrotherapy was the least utilized (58% never used it) (Fig. 1).

Regarding exercise, balance training was the most used, followed by strength exercises; treadmill and bicycle training were the most minor (5.90% never use it). About specific neurological techniques, the task-oriented approach was the most used; on the other hand, the constraint-induced movement therapy (CIMT) was the least used technique (55.90%) never used it. The respondents did not generally use advanced technologies or other techniques such as music therapy or mindfulness in physiotherapy stroke rehabilitation in Catalonia.

#### 3.1.2 Descriptive Analysis of the Method Approaches in Physiotherapy

Participants were asked: “During the rehabilitation of people with stroke, do you follow any of these specific approach methods? (non-specific approach, Basale stimulation (BS), neurocognitive therapy, neurodevelopment therapy and Proprioceptive Neuromuscular Facilitation (PNF))”. The chosen approach should represent the primary focus of your clinical practice. The results showed that 57.60% of the subjects indicated that they do not adhere to a specific method; instead, they employ various approaches depending on the patient or the stage of the stroke, often using a combination of methods (Table 2).

**Table 2.** **Frequencies of the different methods used in stroke rehabilitation**.

Methods	Frequency	Percentage
Non-Specific method	68	57.60%
Basale Stimulation	15	12.70%
Neurocognitive therapy	14	11.90%
Neurodevelopmental therapy	11	9.30%
PNF	10	8.50%
Total	118	100.00%

PNF, Proprioceptive Neuromuscular Facilitation.

Descriptive analysis of the specific and non-specific approaches with post stroke phases, settings, and years of experience. Coded variables and Description of the frequencies.

There were no significant differences between phases regarding the utilization of a specific approach or not (χ^2^ = 0.53; *p*-value = 0.76). Additionally, no significant differences were found in utilizing specific or non-specific approaches in public and private settings (χ^2^ = 2.82; *p*-value = 0.24). Similarly, no differences were observed between years of experience and the approaches they employ (χ^2^ = 0.81; *p*-value = 0.66).

There was no association between stroke stages and the methodological approach used, as most respondents did not employ a specific method. However, during the subacute and chronic phases following a stroke, BS and neurocognitive therapy appear to be the second most used approaches.

Similarly, there was no association between public or private settings and the methodological approach used. Both settings predominantly do not employ a specific method. However, in private settings, BS, neurocognitive therapy, and neurodevelopment therapy were more frequently utilized compared to public settings. In contrast, PNF was more used in public than in private settings.

Most respondents indicated that they did not employ a specific method regardless of their years of experience. However, respondents with over ten years of experience tended to utilize BS, neurocognitive therapy, neurodevelopmental therapy, and PNF more frequently. Conversely, respondents with 0 to 2 years of experience tended to use neurodevelopmental therapy and PNF (Table 3).

**Table 3.** **Frequencies of associations between the method and poststroke phases, work setting and years of experience of the physiotherapists**.

		Non-Specific method	Basale Stimulation	Neurocognitive therapy	Neurodevelopment therapy	PNF	Total
Phases poststroke							
	Acute	0	1	0	1	1	3
	Acute/Subacute	4	0	0	2	1	7
	Subacute	16	0	1	3	2	22
	Subacute/Chronic	23	10	7	3	5	48
	Chronic	15	4	4	2	1	26
	Acute/Subacute/Chronic	10	0	2	0	0	12
	Total	68	15	14	11	10	118
Years of experience							
	0–2 years	12	0	2	3	4	21
	3–5 years	19	5	2	2	1	29
	6–9 years	11	3	1	2	2	19
	>10 years	26	7	9	4	3	49
	Total	68	15	14	11	10	118
Work Setting							
	Public	24	1	3	2	5	35
	Private	31	9	9	5	2	56
	Both	13	5	2	4	3	27
	Total	68	15	14	11	10	118

#### 3.1.3 Describing Conventional Physiotherapy Across Open Question

**Fig. 2.**
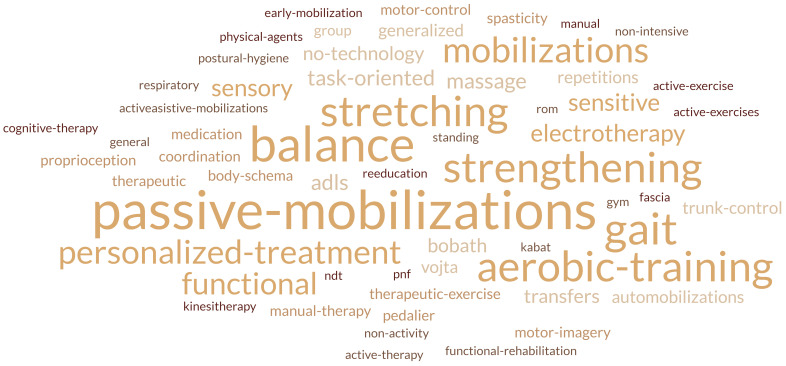
**Word cloud generated from responses to the open-ended question ‘What is conventional physiotherapy?’**. The size of each word corresponds to its frequency

In the open question, participants were asked, “In your opinion, what do you consider as conventional therapy for people who have suffered a stroke?”. The survey findings indicated that several respondents were unsure of what conventional physiotherapy includes. However, most respondents agreed that it includes passive mobilizations, stretching, strength exercise, balance, and gait training. Other therapies mentioned included methods such as Bobath, Votja, and PNF (Fig. 2).

## 4. Discussion

To the best of our knowledge, this is the first study that pretends to know what conventional physiotherapy within stroke rehabilitation includes, specifically in the context of Catalonia, Spain. The term “conventional physiotherapy” currently lacks standardization, rendering it challenging to discern the specific methods and approaches this broad category encompasses. In the study, the mission of physiotherapists who participated in the survey was to identify and categorize their rehabilitation practices in people with stroke.

Regarding the use of classic physiotherapy techniques, passive mobilization emerges as the most used in stroke rehabilitation in Catalonia, followed closely by stretching. Balance and strength exercises take precedence in exercise techniques, followed by therapeutic physical exercise. Within neurologically specific techniques, the task-oriented approach claims the top spot, trailed by bimanual therapy, motor imagery, mirror therapy, and intensive therapy. On the contrary, CIMT ranks as the least utilized approach. These findings align with Arienti *et al*.’s 2022 study [17], noting the underutilization of CIMT despite the evidence supporting its efficacy compared to other rehabilitation approaches on the recovery from motor impairment and motor function but not in disability. Physiotherapists may not employ this technique probably because of the specific type and intensity of exercise required and patient inclusion criteria, requiring some degrees of active extension in the wrist and fingers, minimal pain or spasticity, and a high level of compliance with the rehabilitation treatment [22].

According to our study, physiotherapists in Catalonia rarely used advanced technologies (such as virtual reality) despite the extensive growing evidence of their use [14]. In our view, the lack of investment in advanced technologies in public settings stems from a reticence to invest in costly rehabilitation equipment designed for individual use. Other techniques, such as music therapy or mindfulness, are not widely used, possibly due to the need for more evidence and results from these interventions [23, 24] or the lack of training in these disciplines among physiotherapists.

Concerning the analysis of the method approaches in conventional physiotherapy, our results showed that physiotherapists agreed they do not use a specific method during their interventions, as corroborated by a new revision [17]. Therefore, our results have shown the downward usage trends observed in recent years and manifest the change in the therapeutic approach to manage people with stroke [25, 26], due to most of the Catalan physiotherapists have already left behind the exclusive use of classic methods such as the Bobath, Brunnström, Rood method and PNF [26], following the recommendations of the new clinical practice guidelines [10, 19].

Our results showed that BS, neurocognitive therapy, and neurodevelopment therapy are more used in private settings than in public ones. In our opinion, this greater presence may be partly because private centers operate for business reasons, following a more specific approach or methodology, differentiating themselves from the others. Furthermore, private centers have the time necessary to develop these techniques. On the other hand, in public settings, as mentioned, they may follow the guidelines of the clinical practice guides, so they have more flexibility when using one technique or another. Another possible explanation for the lack of a specific method in the public setting is the limited time available per patient, influenced by the institution’s organization and the shortage of human resources. There significant differences between public and private settings regarding the time allocated per session for a patient. Physiotherapists in private settings typically spend one hour or more with each patient; the Spanish Neurorehabilitation Society Clinical Practice Guide [27] recommends a minimum of 45 minutes, five days a week. In contrast, those working in the public sector generally allocate between 15 and 45 minutes per stroke patient. Moreover, they often conduct more group sessions than individual sessions.

When examining the various stages of stroke and the approaches employed, our results showed a notable absence of substantial differences, consistent with the systematic review conducted by Arienti *et al*. in 2022 [17]. In contrast, the differences depend on whether they go to a private or public setting. In Catalonia, people with chronic stroke do not usually go to public settings to continue their rehabilitation; they only undergo routine checks in Primary Care. To continue their treatment, usually they are typically obligated to seek services at a private center [28].

One limitation of this study is the data collection instrument. Since no previous studies explored the proposed objective, a questionnaire was developed by experts in neurological physiotherapy. However, it exhibits certain methodological biases inherent to its design. Although the questionnaire underwent a pilot test, it is not a validated or standardized assessment tool. As a result, variations in participants’ interpretations may have occurred, particularly in the section addressing therapeutic approaches and techniques. Another limitation of the study is the response bias, as we do not have a real number of the active neurological physiotherapy community in Catalonia to compare with; therefore, the response rate percentage is unknown.

## 5. Conclusions

The study concludes that physiotherapists in Catalonia generally do not rely on a specific method or technique; instead, they use a combination of them. However, the three most used techniques or approaches are the task-oriented approach, balance training and strength training. Conversely, the three least (or never) used techniques are acupuncture, meditation, and dry needling. In regard to participants’ opinions on what conventional physiotherapy is, the three more linked concepts were passive mobilizations, stretching and strength exercise.

Consequently, we cannot definitively conclude what conventional physiotherapy includes due to the heterogeneity of answers collected in this survey and the huge variety of existing techniques and approaches. Due to this uncertainty, it is advisable to explicitly state the techniques and methods that encompass conventional physical therapy in all scientific studies of this nature.

## Availability of Data and Materials

The datasets generated during the current study are available from the first author on reasonable request.

## Supplementary Material

Supplementary material associated with this article can be found, in the online version, at https://doi.org/10.31083/RN37316.
