# Supplementary material for: Mapping Physiotherapy Approaches for Stroke Survivors in Catalonia: A Cross-Sectional Study
Source: Rev Neurol. 2025 Jun 16;80(5):37316. doi: 10.31083/RN37316 (PMC12231508; doi:10.31083/RN37316)
Supplement: Supplementary file 1 [file 1576-6578-80-5-37316-s1.zip › Supplementary Material 2.docx]

**Questionnaire**

1. Do you agree to participate in this survey?

*Mark only one oval.*

Y N

**Demographic Data**

1. Gender*

*Mark only one oval.*

Male

Female

Non-binary

1. Age *

*Mark only one oval.*

21-30

31-40

41-50

51-65

≥ 66

1. Region*

*Mark only one oval.*

Barcelona Girona

Lleida Tarragona

1. Year of Completion of Degree in physiotherapy *
2. Do you have specific training in neurological physiotherapy? *

*Mark only one oval.*

Yes

No *Skip to question 8*

**Specific training in neurological physiotherapy**

1. Do you have specific training in neurological physiotherapy?*

*Mark only one oval.*

Continuing education courses

Postgraduate program

Master's degree

PhD or PhD student

**Experience in neurorehabilitation**

1. Years of experience in the field of neurorehabilitation? *

*Mark only one oval.*

0-2 years/years

3-5 years/ years

6-9 years/years

≥ 10 years/years

1. Select the option that best suits your current work environment. *

*Mark only one oval.*

Public

Private

Both

1. Select the option that best suits the type of institution where you work. *

*Select all that apply.*

Hospital

Rehabilitation Center, medical clinics, Mutual insurance centers

Home Rehabilitation

Healthcare and social care center

Other:

1. In your work environment, do you work in a multi/inter/transdisciplinary team? *

*Mark only one oval.*

Yes

No *Skip to question 13*

**Disciplinary team**

1. Select the professionals who are part of your team. *

*Select all that apply.*

Occupational Therapist

Speech Therapist

Psychologist (clinic, neuropsychologist)

Orthopedic Technician

Clinic or rehabilitation assistants

Nursing

Rehabilitation physician

Neurologist

Social Worker

Surgeon

Other:

**Therapeutic intervention**

1. Weekly, how many physiotherapy sessions do you usually carry out with patients who have suffered a stroke? *
2. Generally, the post-stroke patients you treat are in the phase: *

*Select all that apply.*

Hyper-Acute (<24 hours/hours)

Acute (>24 hours/hours, <7 days/days)

Early subacute (>7 days ≤ 3 months) /Early subacute (>7 days ≤ 3 months)

Late sub-acute (>3 months ≤6 months) / Late sub-acute (>3 months ≤6 months) Chronic (> 6 months) / Chronic (> 6 months)

1. In the rehabilitation of stroke patients, you are governed by one of this specific type of approach (represents the main focus in your clinical practice)*

*Mark only one oval.*

Neurodevelopment therapy (ex. Bobath)

Neurocognitive therapy (ex. Perfetti, Affolter) PNF (Proprioceptive neuromuscular facilitation) (ex. Kabat) Vojta Method

Basale Stimulation

I do not use any specific approach

Other:

1. Do you use any of these passive techniques in your intervention? *

*Mark only one oval per row.*

|  | *Nothing* | *Little* | *Occasionally* | *Frequently* | *Always* |
| --- | --- | --- | --- | --- | --- |
| *Stretching* |  |  |  |  |  |
| *Passive Mobilizations* |  |  |  |  |  |
| *Electrotherapy (US, TENS…)* |  |  |  |  |  |
| *Massotherapy* |  |  |  |  |  |
| *Dry Needling* |  |  |  |  |  |
| *Mindfulness* |  |  |  |  |  |
| *Meditation* |  |  |  |  |  |
| *Acupuncture* |  |  |  |  |  |

1. Do you use any of these active techniques in your intervention? *

*Mark only one oval per row.*

|  | *Never* | *Rarely* | *Occasionally* | *Frequently* | *Always* |
| --- | --- | --- | --- | --- | --- |
| *Task- Oriented Training* |  |  |  |  |  |
| *Mirror Therapy* |  |  |  |  |  |
| *Bimanual Therapy* |  |  |  |  |  |
| *Intensive Therapy* |  |  |  |  |  |
| *CIMT (constrain induced movement therapy)* |  |  |  |  |  |
| *Motor Image* |  |  |  |  |  |
| *Advanced Technologies (Virtual reality, serious games…)* |  |  |  |  |  |
| *TICS (Apps)* |  |  |  |  |  |
| *Treadmill/ Static bike* |  |  |  |  |  |
| *Music Therapy* |  |  |  |  |  |
| *Balance Training (ex. CORE, unstable surfaces BP and SD)* |  |  |  |  |  |
| *Strength exercises (with or without machines)* |  |  |  |  |  |
| *Therapeutic exercise* |  |  |  |  |  |

1. Do you do individual therapy?*

*Mark only one oval.*

Yes

No *Skip to question 21*

**Individual therapy**

1. How do you describe the individual sessions you perform? / *

*Mark only one oval.*

Synchronous (always accompanying the patient)

Asynchronous (supervising the patient) Both

1. How long do your rehabilitation sessions usually last per patient? *

*Select all that apply.*

≤ 15 min

15-30 min

30-45 min

1 hour

- - 1 hour

**Therapy group**

1. Do you do group therapy?*

*Mark only one oval.*

Yes

No *Skip to question 25*

1. How do you describe the group sessions you carry out? /How would you describe the *

group sessions that you do?

*Mark only one oval.*

Synchronous (always accompanying the patient)

Asynchronous (supervising the patient) Both

1. How long do your group rehabilitation sessions usually last ? *

*Select all that apply.*

≤ 15 min

15-30 min

30-45 min

1 hour

- - 1 hour

1. How many patients do you have per group? *

**Therapy**

1. How many sessions per week do most of your patients usually perform? *

*Select all that apply.*

1 session

2-4 sessions

≥ 5 sessions

1. Do the patients you attend have a limit on the number of sessions they can perform? (a maximum set by a professional/entity, time limitation,..) *

*Select all that apply.*

Y N

**Extra question**

1. In your opinion, what do you consider conventional physiotherapy?
